# Abscopal Response in Metastatic Melanoma: Real-World Data of a Retrospective, Multicenter Study

**DOI:** 10.3390/cancers14174213

**Published:** 2022-08-30

**Authors:** Luc Ollivier, Charles Orione, Paul Bore, Laurent Misery, Delphine Legoupil, Jean-Christophe Leclere, Anne Coste, Gilles Girault, Iona Sicard-Cras, Clemence Kacperek, Francois Lucia, Dinu Stefan, François Thillays, Emmanuel Rio, Paul Lesueur, Christian Berthou, Dominique Heymann, Stéphane Champiat, Stéphane Supiot, Loig Vaugier, William Kao

**Affiliations:** 1 Department of Radiation Oncology, Institut de Cancérologie de l’Ouest (ICO), 44800 Saint-Herblain, France; 2 Département de Médecine Interne et Pneumologie, Centre Hospitalo-Universitaire de Brest, Université de Bretagne Occidentale, 29609 Brest, France; 3 Oncology Department, Institut de Cancérologie de l’Ouest (ICO), 44800 Saint-Herblain, France; 4 Service de Dermatologie, CHU, 2, Avenue Foch, 29200 Brest, France; 5 Laboratoire sur les Interactions Épithéliums-Neurones (LIEN-EA4685), Université de Bretagne Occidentale, 29200 Brest, France; 6 Service d’ORL et de Chirurgie Cervico-Faciale, Hôpital Morvan, Centre Hospitalo-Universitaire de Brest, Université de Bretagne Occidentale, 5, Avenue Foch, 29200 Brest, France; 7 Département de Maladie Infectieuses, Centre Hospitalo-Universitaire de Brest, Université de Bretagne Occidentale, 29609 Brest, France; 8 Unicancer, Comprehensive Cancer Center F. Baclesse, Medical Library, F-14000 Caen, France; 9 Service de Pédiatrie, Hôpital Morvan, Centre Hospitalo-Universitaire de Brest, Université de Bretagne Occidentale, 5, Avenue Foch, 29200 Brest, France; 10 Département of Radiation Oncology, Centre Hospitalier de Cornouaille, 14 Avenue Yves Thepot, 29000 Quimper, France; 11 Radiation Oncology Department, Centre Hospitalo-Universitaire de Brest, Université de Bretagne Occidentale-Hôpital Morvan-2 Avenue Foch, 29200 Brest, France; 12 Centre Guillaume le Conquérant, Radiation Oncology Department, 76600 Le Havre, France; 13 U1227 B Lymphocytes & Autoimmunity, University of Brest, INSERM, IBSAM, 29200 Brest, France; 14 Nantes Université, CNRS, UMR6286, US2B, 44300 Nantes, France; 15 Department of Oncology and Metabolism, University of Sheffield, Sheffield S10 2RX, UK; 16 Département d’Innovation Thérapeutique et des Essais Précoces (DITEP), Gustave Roussy, Université Paris Saclay, 94805 Villejuif, France

**Keywords:** radiotherapy, immunotherapy, melanoma, metastasis, treatment combination

## Abstract

**Simple Summary:**

An abscopal response (AR) is a rare phenomenon defined as a distant response outside of the radiation field. It opens up the perspective of “in situ” vaccination of cancer. This phenomenon is rare and its mechanisms are unknown. In metastatic melanoma (MM), the approach regarding the efficacy of immunotherapy is to not use immunotherapy as a tool for enhancing radiation response but rather as one that needs to be integrated into immunotherapy to potentiate the specific effects of immunotherapy. The aim of our retrospective study was to investigate the incidence of the AR and its impact on therapeutic outcomes in a homogeneous population of patients with MM and a control group, to identify the factors associated with the AR. AR in metastatic melanoma seems highly prognostic of overall survival although it is a rare phenomenon. Factors associated with AR have been identified.

**Abstract:**

Objective: To evaluate the incidence of the abscopal response (AR) in patients with metastatic melanoma requiring palliative radiotherapy (RT). Patients and methods: Patients treated for metastatic melanoma between January 1998 and February 2020 in four oncology departments were screened. Patients with progression under immune checkpoint inhibitors or without ongoing systemic treatment, and requiring palliative RT were considered. The AR was defined as an objective response according to RECIST and/or iRECIST for at least one non-irradiated metastasis at distance (≥10 cm) from the irradiated lesion. Primary endpoint was the rate of AR. Secondary endpoints were overall survival (OS), progression-free survival (PFS), local control (LC) of the irradiated lesion, and toxicity as assessed by CTCAE v5. Results: Over the period considered, 118 patients were included and analyzed. Fifteen patients (12.7%) had an AR. With a median follow-up of 7.7 months (range, 0.2–242.2), median OS and PFS after RT were significantly longer in patients with an AR compared to those without: 28 vs. 6.6 months (*p* < 0.01) and not reached vs. *3*.2 months, respectively. No grade ≥2 toxicity was reported. Patients who developed an AR were more likely to be treated with immunotherapy (93.3% vs. 55.9%, *p =* 0.02). In multivariate analysis, they had a higher number of irradiated metastases treated concomitantly (HR = 16.9, *p* < 0.01) and a higher rate of mild infections during RT (HR = 403.5, *p* < 0.01). Conclusions: AR in metastatic melanoma seems to be highly prognostic of overall survival, although it is a rare phenomenon. It may be promoted by multiple concomitant treatments with RT and immunotherapy and by acute inflammatory events such as infection.

## 1. Introduction

Radiotherapy can be used to convert a tumor into an “in situ’’ vaccine [1]. The mechanisms of this concept are related to the immune system: when focal radiation therapy is applied to the tumor and the tumor microenvironment, it induces immunogenic cell death, a type of cell death which can be recognized by the immune system. This process can generate an immune response that contributes not only to the in-field response, but also to the out of field response and in occasional situations it has been described as an abscopal response. However, the abscopal effect is very rare because tumors are established, with metastases and many mechanisms of immune evasion, and it is extremely difficult for radiotherapy to one site to induce an immune response. However, in combination with immunotherapy, the situation can be different [2]. The problem is shifting the balance of the mixed signals from immunosuppressive to pro-immunogenic and trying to either mitigate the negative effect of radiation or enhance the positive effect of radiation [3]. This challenge has been explored in numerous preclinical models, with some translated to clinical practice. In metastatic melanoma, the approach regarding the efficacy of immunotherapy is to not use immunotherapy as a tool for enhancing the radiation response, but rather as a tool that needs to be integrated into immunotherapy to potentiate the specific effects of immunotherapy. With the emergence of immune checkpoint inhibitors of programmed death-1 (PD-1), programmed death-ligand 1 (PD-L1) and cytotoxic T-lymphocyte-associated protein 4 (CTLA 4), the 5-year overall survival (OS) rate of patients with metastatic melanoma (MM) has dramatically improved in the last decade, reaching 50%, as well as with 20% of long responder patients [4,5,6]. Large tumor mutational burden and/or the immune-mediated patient-dependent environment may partly explain such significant outcomes.

Simultaneously, the interest in one of the holy grails of radiation oncology was reborn. Does the abscopal response (AR) exist, i.e., could the local radiation-induced tumor response generate a distant, out-of-field tumor response? Since the first description of the AR in 1953 by Dr. RJ Mole [7], only 46 cases of AR have been reported between 1953 and 2016 [8] and 24 cases in combination with immunotherapy [9]. A patient-level data meta-analysis for the predictors of response was performed in order to report data on progression free-survival (PFS), distant metastases, and overall survival with the hypothesis that certain clinical covariates may predict the survival of patients with an abscopal response. Among 67% of abscopal responses in non-squamous cell lung cancer, kidney cancer, melanoma, lymphoma, and hepatobiliary cancer, there were no clinical predictors of the duration of response or survival, as well as a lack of control group [10]. Additionally, many trials have been conducted to reproduce this phenomenon with disappointing results. Nowadays, the AR remains one of the most active areas of research in oncology [11]. Great interest is given to the mechanisms and trigger factors relative to the AR, especially regarding specific radiotherapy (RT) modalities.

MM is recognized as one of the most immune-sensitive oncologic diseases and patients with MM may thus be the most likely to show an AR [12]. The aim of our study was to investigate the incidence of AR and its impact on therapeutic outcomes in a homogeneous cohort of patients with MM. Factors associated with AR were also identified.

## 2. Patients and Methods

### 2.1. Patient Population

Retrospective data extraction was performed between January 1998 and February 2020 at the University Hospital in Brest (France), the Regional Hospital in Quimper (France), the Francois Baclesse Cancer Center in Caen (France), and the Institut de Cancérologie de l’Ouest (Saint-Herblain, France). All consecutive patients with MM and requiring palliative RT were considered. Inclusion criteria were: histologically proven MM, disease progression under immunotherapy according to the Immune Response Evaluation Criteria in Solid Tumors (iRECIST) or disease progression without systemic treatment (i.e., patients unfit for immunotherapy), RT for at least one primary or metastatic site, and the presence of at least one non-irradiated metastasis which was (i) ≥10 cm away from the irradiated target volume and (ii) assessable according to iRECIST criteria. A cut-off of 10 cm was arbitrarily set to avoid biases arising from potential low-dose radiation responses. Exclusion criteria were: initiation of a new systemic treatment, irradiation of brain metastases, and previous or concomitant treatment with BRAF inhibitors.

All RT schedules were accepted. The RT-target irradiation volume was defined as the planning target volume (PTV) covered by at least 95% of the prescribed dose. The following patient characteristics were recorded at the beginning of the RT: Eastern Cooperative Oncology Group Performance Status (PS), American Joint Committee on Cancer 8th edition (AJCC 8th) TNM [13], RT total dose, RT dose per fraction, RT duration, the localization of target volumes, number of irradiated sites treated simultaneously, modalities and date of the first day of the last systemic treatment, the continuation of the last systemic treatment, and primary and secondary resistance status. Primary resistance was defined as the absence of clinical response after initial PD-1/PD-L1 blockade. Secondary resistance refers to progression while on therapy despite an initial response, or unresponsive to re-initiation of checkpoint blockade with the need for local symptomatic treatment such as RT [14]. 

Individual immune-related events (irE) were defined as acute events occurring during RT only: documented infectious episodes, clinical symptoms of acute inflammation (redness, heat, swelling, pain), and/or biological inflammatory syndrome with elevation of inflammation parameters including at least C-reactive protein (CRP) > 30 mg/L). Clinical acute inflammation was classified as (i) light clinical inflammation and CRP <40 mg/L, (ii) moderate clinical inflammation and CRP <40 mg/L, and (iii) severe clinical inflammation and CRP >50 mg/L [15]. 

Usual follow-up after RT in participating centers is performed by an onco-dermatologist every 3 months and a radiation-oncologist every 6 months. Clinical examination, blood samples, and either a computed tomography (CT) or a 18F-Fluorodeoxyglucose positron emission tomography with CT (^18^F-FDG-PET/CT) are performed.

### 2.2. Outcome

The primary endpoint was the rate of patients developing an AR following RT. An AR was defined as an objective response rate for at least one non-irradiated metastasis, namely, either complete response (CR) or partial response (PR) according to iRECIST [16,17]. All patients with a suspected AR were confirmed with a double reading by two radiation oncologists. Secondary outcomes were local control (LC) of the irradiated lesion, overall survival (OS), progression-free survival (PFS), and toxicity as assessed by the Common Terminology Criteria for Adverse Events version 5 (CTCAE v5) classification [18]. LC was defined as complete response (CR) or partial response (PR) in the irradiated lesion as per iRECIST. In specific cases of new or re-growth of existing lesions within or at the margin of the PTV, the lesion was considered locally progressive. If progression was confirmed at the next assessment, the date of progression assigned was the earlier date when progression was first suspected. Distant progression was defined as the appearance of new lesions outside the PTV.

### 2.3. Statistical Analysis

The chi-square test or Fisher’s exact test was used to analyze and compare patients’ clinical characteristics at baseline between groups for categorical variables. Student *t*-test or the Wilcoxon test was used for continuous variables, according to their distributions. PFS and OS were defined as the time between the first day of RT and either local, regional, or distant progression or death, respectively. Survival (PFS, OS) was described by means of Kaplan–Meier curves and compared using log-rank tests in univariate analysis.

OS and PFS were analyzed with a multivariable Cox model considering AR as a time-dependent binary variable, since its value changed over time among patients who experienced an abscopal effect.

A secondary analysis used another Cox model to identify risk factors for developing AR over time. For the dosimetric variables, the ROC curve was built to identify the best value associated with the occurrence of AR within the first 30 days after the beginning of RT. 

For each variable and each model, the proportional hazard hypothesis and the log-linearity for continuous variables were assessed using Schoenfeld residuals and graphical visualization, respectively.

Statistical analyses were performed using R software-© 2009–2019 RStudio, Inc.

## 3. Results

Overall, 118 patients treated with RT for melanoma between January 1998 and February 2020 in four French oncology institutions were included. Median age at the beginning of RT was 66.5 (range, 23.5–100.1). PS was ≤1 in 70.4% patients (Table 1).

### 3.1. Population Characteristics

Sixty-seven percent of patients (*n* = 80) received concomitant antiPD1 +/− antiCTLA4 immunotherapy during RT, including nivolumab (*n* = 47), pembrolizumab (*n* = 16), ipilimumab (*n* = 11), and nivolumab + ipilimumab (*n* = 6). Median time between the first injection of immunotherapy and RT was 2.9 months (range, 0.5–47 months). Patients had primary and secondary resistance to immunotherapy before RT started in 42.5% and 57.5% of cases, respectively (Table 1). The median delivered dose of RT was 30 Gy (range, 6.5–60 Gy). Median dose per fraction was 4 Gy (range, 2–20 Gy). Fifteen patients were treated with stereotactic body RT (SBRT) and the median dose per fraction was 9 Gy (range, 8–20). Median duration of RT was 10 days (range, 1–63). Twenty-two patients (18.6%) had ≥2 simultaneously irradiated metastases. The metastatic sites irradiated were bone lesions (37.3% of patients), lymph nodes (23.7%), and skin metastases (19.5%). Conformal RT with linear accelerators was used for all patients. No cases of severe grade 2+ acute or late toxicity were reported. IrE occurred in 16 patients (13.5%) during RT and were mainly mild (Table 2).

### 3.2. Survival Analysis

**Figure 1:**
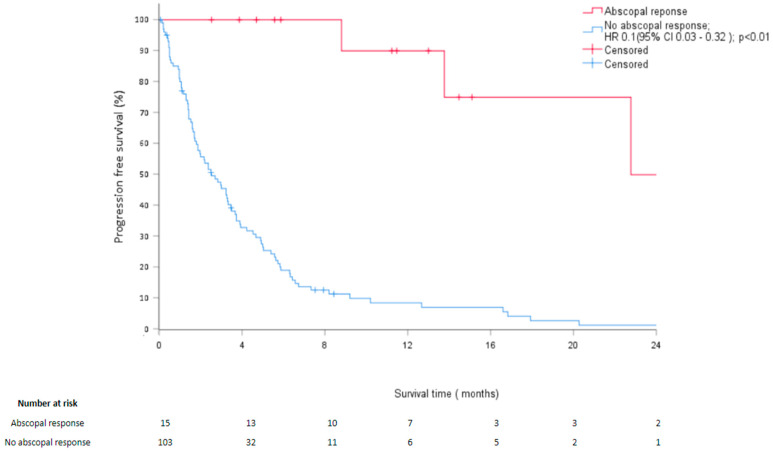
Kaplan- Meier estimates of progression-free survival at 24 months depending on the occurrence of an abscopal response.

An AR was observed after RT in 15 patients (12.7%) after a median time following RT of 1.7 months (range, 0.2–3.6) and persisted over a median time of 11.9 months (range, 4.2–68.4) (Table 2). With median follow-up of 7.7 months (range, 0.2–242.2), LC or stable disease for the irradiated lesions was obtained in 103 patients (87.3%) (Table 3). Median PFS after RT was 3.6 months (95% CI: 2.7–4.4). Median PFS was 3.2 months for patients without AR, whereas it was not reached in cases of AR (Figure 1). In multivariate analyses, the occurrence of AR was associated with increased PFS (*p* < 0.01) (Table 4). Median OS after RT was 8.5 months (95% CI: 4.3–12.6).

**Figure 2:**
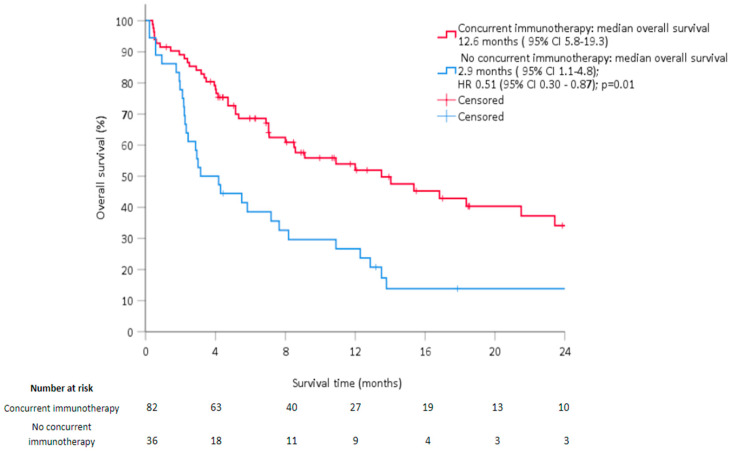
Kaplan–Meier estimates of overall survival at 24 months depending on the administration of immunotherapy.

**Figure 3:**
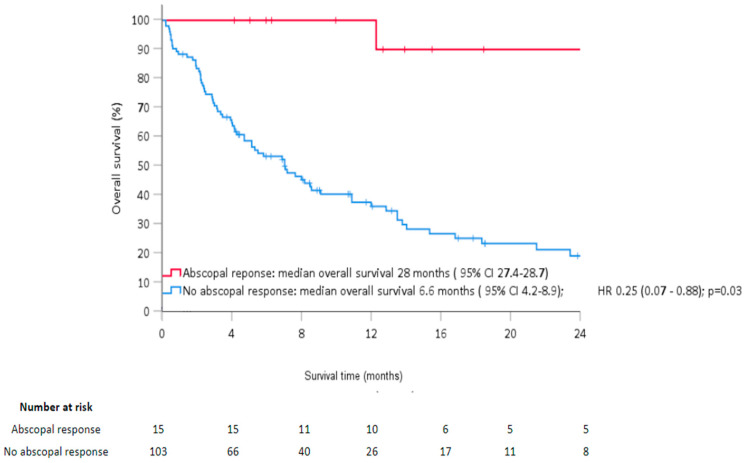
Kaplan–Meier estimates of overall survival at 24 months depending on the occurrence of abscopal response.

Among the variables tested in multivariate analysis, the occurrence of an AR (HR = 0.19, *p =* 0.007) and concomitant immunotherapy (HR = 0.51, *p =* 0.01) significantly improved OS (Table 5), whereas a poor performance status (WHO 2–3) was negatively correlated with OS (*p* < 0.01). OS was significantly longer in patients who received immunotherapy [12.6 (95%CI: 5.8–19.3) versus 2.9 months (95%CI: 1.1–4.8)] (Figure 2) and in those who developed an AR compared to those who did not (28 months (95% CI: 27.4–28.7) versus 6.6 months (95% CI: 4.2–8.9), (*p* < 0.01)) (Figure 3).

### 3.3. Abscopal Risk Factors

In multivariate analysis, multiple (≥2) irradiated metastases (HR 16.85 (95% CI 2.16–131.49) *p* < 0.01) and concomitant irE (HR 403.45 (95% CI 13.83–11769.39), *p* < 0.01) were significantly associated with the occurrence of an AR (Table 6).

**Figure 4:**
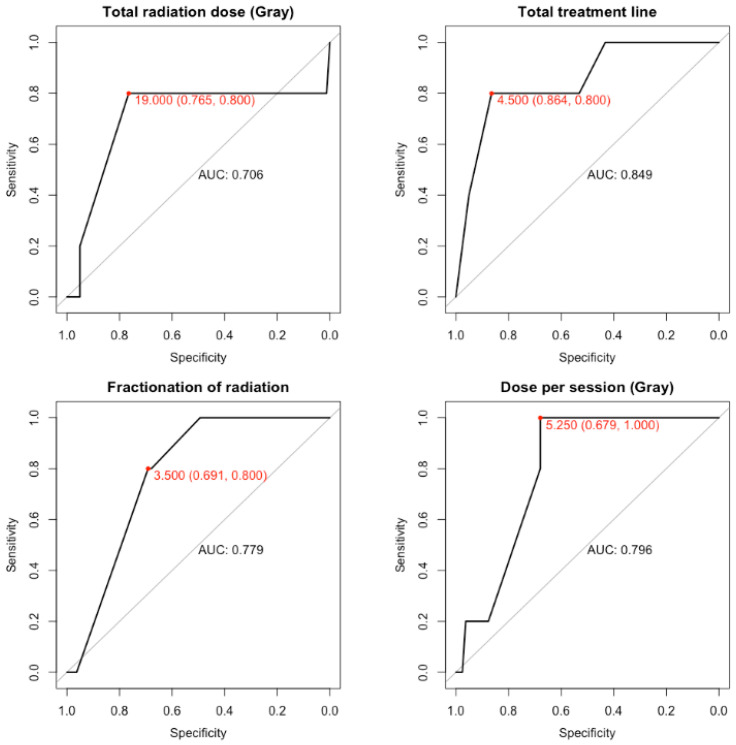
ROC curves for the dosimetric parameters. *AUC = Area under the curve*.

For dosimetric parameters, the optimal values associated with a higher chance of developing an abscopal response at 30 days was estimated at 19 Gy (AUC = 0.706) delivered in 3.5 fractions (AUC = 0.779) of 5.25 Gy (AUC = 0.796) over 4.5 days (AUC = 0.849) (Table 7,Figure 4).

## 4. Discussion

In our multicenter cohort including 118 patients with MM treated with palliative RT, 15 patients (12.7%) developed an AR. In comparison, other studies found evidence for AR in 34.3% (range, 18–63%) of cases in an analysis of 10 nonrandomized studies of metastatic melanoma patients treated with RT and ICI between 2014 and 2019 [19]. Our study is of moderate size compared to others given the eligibility criteria and the strict definition of an abscopal response.

The occurrence of AR in our study was associated with significantly improved oncologic outcomes, including OS rates in multivariate analysis. By definition, an AR correlates with effective distant control; interestingly, it also resulted in better OS rates. Our results are in accordance with the literature: in a prospective study that aimed to induce an abscopal effect in 41 stable or progressing patients treated with systemic treatment for solid tumors, the median OS was 20.98 months (95% CI 11.05–30.96) in abscopal responders versus 8.33 months in others (95% CI 5.03–13.29). This study, however, did not include patients with metastatic melanoma, but mostly lung and breast cancer [20].

With a median follow-up of 7.7 months, median OS (10.2 months) after the occurrence of antiPD1 +/− antiCTLA4 immunotherapy resistance was poor in our cohort, which is in agreement with the literature [21]. Immunotherapy was continued concomitantly with RT in 63% of patients despite progression under such treatment. Although this attitude may appear contradictory, it is often justified by i) the addition of RT for the most aggressive or painful lesions, ii) the possibility of a delayed response under immunotherapy, and iii) the potential synergy between immunotherapy and RT [22]. Patients treated with RT and immunotherapy had significantly better survival outcomes compared to RT alone, with a median OS of 12.6 months (95%CI: 5.8–19.3) versus 2.9 months (95%CI: 1.1–4.8) (*p* < 0.01). These observations—also coherent with the literature—corroborate such a frequently adopted strategy [22]. The use of ipilimumab [23] has been associated with the AR, but the immunotherapy sequence with respect to RT needs to be discussed in further prospective studies to promote an abscopal response. The administration of antiPD1 before irradiation seems to abrogate systemic immunity and increase the radiosensitivity of CD8+ T cells, while tumor control is improved when antiPD1 is given after RT [24].

Such AR-associated improved outcomes justify investigating how AR might be provoked. The interplay between immune environment and AR has already been described [25] and numerous protocols have already been employed, such as the use of granulocyte-macrophage colony-stimulating factor (GM-CSF) concomitantly with RT and chemo- or hormonotherapy, SBRT, and immunotherapy [20,26]. In the present study, the occurrence of mild infections was observed more frequently in patients with an AR. To our knowledge, the potential role of infection-driven inflammation as an AR-promoting factor has never been described. Polynuclear neutrophils—whose recruitment and type may have significant antitumor activity as shown in the proof-of-principle trial of GM-CSF and radiotherapy [20,27,28]—could be part of the explanation. However, previous studies (but not in cases of MM) have shown an inverse relationship between RT efficacy and hyperleukocytosis [29]. The role of neutrophils with RT thus remains controversial [30]. One possible explanation may be the induction by RT of type I interferon (IFN-I), which is a critical factor. In irradiated tumors and viral infections, IFN-I is induced by cytosolic DNA that stimulates cGAS to produce cGAMP, leading to STING activation. IFN-I activates conventional dendritic type 1 cells that cross-present the antigens to CD8 T-cells to eliminate the infected or tumoral cells [31].

Activation of the interferon pathway may also come from the dose of radiotherapy and fractionation dose. In fact, in preclinical data regarding 8 Gy, there is more induction of IFN than at higher doses, such as 20 Gy or 30 Gy. Repeating the dose of 8 Gy three times seems to be the optimal formula for activating a type I IFN response and upregulates the expression of MHC-I. [32,33]. At higher doses, further stimulation of DNA damage leads to negative feedback expression of 3-prime repair endonuclease 1 (TREX1), which digests cytosolic DNA and thus reduces the activation of the IFN I response [34]. In a subset analysis of a multicenter clinical trial with matched biopsied pre/post RT plus pembrolizumab, a complete response and partial response were found in patients with a low level of TREX1 and a high level of DNASE1, which is the opposite of TREX1 [35]. In our study, optimal dosimetric parameters for inducing an abscopal response at 30 days may be 19 Gy delivered in 3.5 fractions of 5.25 Gy over 4.5 days. This analysis should be accepted with caution given the small number of patients and the heterogeneity in practices. However, it seems to concur with preclinical findings where hypo-fractionated RT was superior to a single dose of RT in promoting an antitumor immune response with a dose/fraction between 5–7.5 Gy [36] and a number of fractions between 3 and 5 [31].

Among other agents involved in the immune response against external injuries, lymphocytes are less likely to be involved as they are known to be radiosensitive and thus strongly depleted within radiation fields. This understanding has led to the idea that blood should be considered an organ at risk because RT-induced lymphopenia is associated with poorer survival in multiple tumor types [37]. Lung cancer radiotherapy with 60 Gy/30 fractions exposes 99% of circulating blood to more than 0.5 Gy [38] and that has an impact on the efficacy of the patient’s immune system and seems to be associated with low survival. Then, when combining radiotherapy with immunotherapy, it seems to be preferable to use hypo-fractionation, a small field size, and the integral dose.

In contrast, dendritic cells, while particularly stimulated during infections [39], are capable of surviving common radiation doses, making them potential vectors for the distance antitumor response of the RT targets [40,41].

All these hypotheses merit further exploration but are currently not robust enough to be reproducible and need to be documented as accurately as possible. Infection-driven immune-related events can barely be distinguished from treatment-related immune events. As our study was retrospective, the parameters used to monitor inflammation have limitations. Although CRP has relatively good sensitivity for detecting inflammatory processes, it remains non-specific. Concentrations of 10–40 mg/l are generally found in cases of moderate inflammation or viral infections, and typically rise to 50–200 mg/l in cases of severe inflammation or bacterial infections [15], without any clear cut-off [42]. Small increases, between 3 and 10 mg/l, are also found in cases of obesity, smoking, diabetes, and high blood pressure [43]. It is nevertheless interesting to note that in current practice, any infection—from mild to severe—causes RT delay because of the potentially harmful effects of radiotherapy in this context [44], whereas in our study, such events did not seem to induce any increased toxicity and may have even promoted an AR with RT.

A number of irradiated sites ≥ 2 at the same time also appeared to be a favorable factor for the AR in our study, as suggested in the literature [45], but not the type of irradiated anatomical structure. Amplifying the number of tumor antigens and the improved response to immunotherapy following multisite RT has already been reported [46]. In this study, all patients were metastatic, meaning that multi-site irradiation would make possible a higher probability of recognition of neo-antigens in the drainage nodes. In cases of primary tumors, it has been proposed that the draining node should be excluded from the radiation field and only the primary tumor should be irradiated [47], with the hypothesis of being immunized against the primary and obtaining a positive node response. This raises the question of excluding drainage nodes when radiotherapy is performed in combination with checkpoint inhibitors. This is relevant in head and neck cancer regarding the negative results of the JAVELIN trial, in which avelumab plus the standard of care chemoradiotherapy versus chemoradiotherapy alone did not improve PFS [48]. To investigate the importance of lymph nodes, preclinical data show that in immune competent mice with tongue tumors, receiving antiCTLA4 reduces tumor volume, but in the case of sham surgery of the draining node, the effect of the antiCTLA4 is abrogated. Similarly, when the nodes are included in the radiation field treating the tongue tumor, the effect of antiCTLA 4 is abrogated [49].

Our study has several limitations, with a possible selection bias inherent to its retrospective design. First, clinicians do not always report infections occurring during radiation therapy. Second, the small proportion of AR patients makes the analysis less robust but remains consistent with the rarity of the phenomenon. Lastly, the follow-up modalities and retrospective data recording may have influenced the results of the analysis.

## 5. Conclusions

The abscopal response in metastatic melanoma patients seems highly prognostic. Although a rare phenomenon, it may be provoked with multiple concomitant radiation treatments with an acute immune-related event during radiotherapy. A biological substrate is urgently needed to corroborate our hypothesis.

## Figures and Tables

**Table 1:** Baseline and patient characteristics.

Baseline Characteristics	Overall
	(*n* = 118)
Age, median (range) years	66.5 (23.5–100.1)
Sex, men (%)	64 (54.2)
Performance status (%)	
0	36 (30.6)
1	47 (39.8)
2	28 (23.7)
3	7 (5.9)
AJCC 8th stage (%)	
M1A/IV	29 (24.6)
M1B/IV	5 (4.2)
M1C/IV	68 (57.6)
M1D/IV	16 (13.6)
Irradiated metastases (%)	
Abdomen (spleen, liver)	13 (11.0)
Lymph node	28 (23.7)
Skin	23 (19.5)
Bone	44 (37.3)
Lung	4 (3.4)
Breast	2 (1.7)
Muscle	4 (3.4)
RT total dose, median (range) Gy	30 (6.5–60)
RT dose per fraction, median (range) Gy	4 (2–20)
SBRT (*n* = 15), median (range) Gy	9 (8–20)
RT duration, median (range) days	10 (1–63)
Multiple (≥2) irradiated metastases	22(18.6)
Concomitant IT (%)	80 (67.8)
Ipilimumab	11 (13.7)
Nivolumab	47 (58.8)
Nivolumab + Ipilimumab	6 (7.5)
Pembrolizumab	16 (20.0)
Time between the first injection of IT and RT, median (range) months.	2.9 (0.5–47)
Immune-related events	16 (13.5)
Resistance to immunotherapy (%)	
Primary	34 (42.5)
Secondary	46 (57.5)

AJCC 8th stage = American Joint Committee on Cancer 8th edition, Gy = Gray, immune-related events = episodes of infection and symptoms of acute clinical inflammation (redness, heat, swelling, pain, loss of function, and/or biological inflammatory syndrome with the elevation of inflammation parameters including at least C-reactive protein). IT = immunotherapy, RT = radiotherapy, SBRT = stereotactic body radiotherapy.

**Table 2:** Summary of the characteristics of abscopal responders.

Sex	Age	1r or 2r to IT or none	Single (1) Or Multiple (≥2) Co RT plans	Site of RT	RT schedules	RT irE	Co-RT anti-infectious agents 0 no 1 yes	LT Before RT	Timing of irE	iRE category	IT	OS (month)	Time between the start of RT and AR detection (days)
F	84	2	1	Splenic metastasis	3*6Gy	Gastritis Helicobacter Pylori	0		concomitant	light	Pembrolizumab	20	17
F	77	0	2	Skin metastases of the leg	7*6Gy	Erysipelas on the same leg	1		concomitant	moderate	-	12	99
M	53	1	2	Pulmonary nodules	5*12Gy 3*6Gy	infectious pneumonia	1		before	moderate	Nivolumab	16	22
F	61	1	1	Left axillary lymph node	3*6Gy	Left Elbow adenitis. rise of CRP: 30 mg/L	1	630	concomitant	light	Nivolumab	18	12
F	75	2	2	Skin metastases of the right leg	3*5.5Gy	erysipelas and urinary infection	1		concomitant	moderate	Nivolumab	32	24
F	56	1	2	Right axillary lymph node + Skin metastases	3*6Gy	Unknown biological inflammatory syndrome. Leucocytes: 18.35 G/L. Neutrophil count: 15.38 G/L CRP: 130 mg/L	0	1080	concomitant	severe	Nivolumab	16	41
F	73	1	1	Skin metastases of the left leg	4*5Gy	Inflammation axillary node Thyroiditis	1		concomitant	light	Nivolumab	12	44
F	66	1	1	Left axillary lymphnode	4*5Gy	Subcutaneous inflammatory hyper metabolism	0		before	light	Nivolumab	63	25
M	65	2	2	Retrocava lymphnode	4*5Gy	Sigmoiditis	0	950	before	moderate	Nivolumab and Ipilimumab	32	26
M	58	2	2	Oral floor+bone metastasis	10*3Gy	Oral infection	1		concomitant	moderate	Nivolumab and Ipilimumab	31	16
W	86	2	1	Left inguinal adenopathy	3*6Gy	bullous pemphigoid	0	900	concomitant	light	Pembrolizumab	13	21
M	72	1	2	Pulmonary nodules	5*12Gy	Rhinopharyngitis +follicular acnea	0	1300	concomitant	moderate	Nivolumab	23	28
M	67	2	1	Skin metastases	13*3Gy	-	-		-		Ipilimumab	67	19
M	85	2	1	Cervical lymph node	15*3Gy	sarcoidosis	-		-	moderate	Nivolumab	18	57
M	88	2	1	Cervical lymph node	30*2Gy	-	-		-		Nivolumab	28	45

IT = Immunotherapy; irE = immune-related events (immune-related events* = episodes of infection and symptoms of acute clinical inflammation (redness. heat. swelling. pain. loss of function and/or biological inflammatory syndrome with elevation of inflammation parameters including at least C-reactive protein); LT = lymphocytes; M = male W = female; 1r or 2r = primary or secondary resistance; RT = radiotherapy.

**Table 3:** Characteristics for the patients with an abscopal response (AR).

Patient Characteristics	No AR	AR	*p*
	*n* = 103	*n* = 15	
Age, median (range) years	66.4 (23.3-100.1)	66.8 (53.5- 87.8)	0.09
Sex, Women (%)	46 (44.7)	8 (53.3)	0.72
PS (%)			0.45
0	32 (31.1)	4 (26.7)	
1	40 (38.8)	7 (46.7)	
2	26 (25.2)	2 (13.3)	
3	5 (4.9)	2 (13.3)	
AJCC 8th stage (%)			0.10
M1A/IV	24 (23.3)	5 (33.4)	
M1B/IV	3 (2.9)	2 (13.3)	
M1C/IV	60 (58.3)	8 (53.3)	
M1D/IV	16 (15.5)	0 (0.0)	
Irradiated metastases (%)			*0.02*
Abdomen (spleen, liver)	11 (10.7)	2 (13.3)	
Lymph node	22 (21.4)	6 (40.0)	
Skin metastases	19 (18.4)	4 (26.7)	
Bone	43 (41.8)	1 (6.7)	
Lung	2 (1.9)	2 (13.3)	
Breast	2 (1.9)	0 (0.0)	
Muscle	4 (3.9)	0 (0.0)	
RT total dose, median(range) Gy	30 (6.5-60)	30(16.5-60)	0.17
RT dose per fraction, median (range) Gy	4(2.4-20)	4 (2-18)	0.09
	10 (1-63)	11 (2-22)	0.49
RT duration, median (range) days			
Multiple (≥2) irradiated metastases (%)	16 (15.5)	6 (40.0)	*0.05*
Concomitant immunotherapy (%)	66 (55.9)	14 (93.3)	*0.02*
Resistance to immunotherapy			0.96
Primary	28 (42.4)	6 (42.9)	
Secondary	38 (57.6)	8 (57.1)	
RT immune-related events *	3 (3.2)	13 (86.7)	*<0.01*
Local response for the RT-targets **			*<0.01*
Progressive	15 (14.6)	0 (0.0)	
Stable disease	58 (56.3)	2 (13.3)	
Tumor regression	30 (29.1)	13 (86.7)	

AJCC 8th stage = American Joint Committee on Cancer 8th edition; RT = radiotherapy; immune-related events* = episodes of infection and symptoms of acute clinical inflammation (redness, heat, swelling, pain, loss of function and/or biological inflammatory syndrome with elevation of inflammation parameters including at least C-reactive protein); ** RECIST 1.1 and/or iRECIST in case of immunotherapy.

**Table 4:** Univariate and multivariate analyses for progression-free survival.

Demographics	Univariate Analysis		Multivariate Analysis	
	HR	*p* Value	HR	*p* Value
Age (per 1 year increase)	0.99 [0.98; 1.01]	0.41		
Sex, female male	Reference 1.55 [1.03; 2.33]	*0.03*	1.89 [1.21; 2.93]	*<0.01*
**Clinical characteristics**				
Performance status 0	Reference			
1	1.09 [0.67; 1.78]	0.72		
2	1.82 [1.06; 3.13]	*0.03*	2.09 [1.21; 3.63]	*<0.01*
3	1.19 [0.42; 3.40]	0.74		
AJCC 8th stage M1A/IV	Reference			
M1B/IV	0.45 [0.13; 1.49]	0.19		
M1C/IV	0.88 [0.55; 1.42]	0.62		
M1D/IV	1.30 [0.67; 2.51]	0.43		
Number of irradiated metastases One metastasis Multiple metastasis (≥2)	Reference 0.56 [0.32; 0.98]	*0.04*	0.83 [0.47; 1.49]	0.53
RT dose	0.98 [0.97; 1.01]	0.09		
Immunotherapy None Concomitant with RT	Reference 0.51 [0.33; 0.78]	*<0.01*	0.64 [0.40; 1.02]	0.06
Abscopal response No AR AR	Reference 0.08 [0.02; 0.24]	*<0.01*	0.1 [0.03; 0.32]	*<0.01*

AJCC 8th stage = American Joint Committee on Cancer 8th edition; RT = radiotherapy, immune-related events* = episodes of infection and symptoms of acute clinical inflammation (redness. heat. swelling. pain. loss of function and/or biological inflammatory syndrome with elevation of inflammation parameters including at least C-reactive protein).

**Table 5:** Univariate and multivariate analyses for overall survival.

Demographics	Univariate Analysis		Multivariate Analysis	
	HR	*p*-Value	HR	*p*-Value
Age (per 1 year increase)	0.99 [0.98; 1.01]	0.64		
Gender female male	Reference 1.31 [0.84; 2.05]	0.23		
**Clinical characteristics**				
Performance status 0	Reference			
1	1.41 [0.79; 2.51]	0.24		
2	3.05 [1.65; 5.63]	**<0.01**	2.73 [1.46; 5.09]	**<0.01**
3	4.20 [1.44; 12.27]	**0.01**	4.70 [1.59; 13.94]	**<0.01**
AJCC 8th stage M1A/IV	Reference			
M1B/IV	0.63 [0.14; 2.72]	0.53		
M1C/IV	1.57 [0.90; 2.75]	0.11		
M1D/IV	2.20 [1.04; 4.67]	**0.04**	2.45 [0.164; 3.97]	0.63
Number of irradiated metastases One metastasis Multiple metastasis (≥2)	Reference 0.38 [0.18; 0.78]	**0.01**	0.48 [0.23; 1.02]	**0.05**
RT dose	0.98 [0.96; 0.99]	**0.04**	0.98 [0.96; 1.01]	0.11
Immunotherapy None Concomitant with RT	Reference 0.49 [0.31; 0.77]	**<0.01**	0.51 [0.30; 0.87]	**0.01**
Abscopal response	0.19 [0.04; 0.46]	**<0.01**	0.25 [0.07; 0.88]	**0.** **03**

AJCC 8th stage = American Joint Committee on Cancer 8th edition; RT = radiotherapy, immune-related events = episodes of infection and symptoms of acute clinical inflammation (redness, heat, swelling, pain, loss of function, and/or biological inflammatory syndrome with the elevation of inflammation parameters, including, at least, C-reactive protein).

**Table 6:** Risk factors of abscopal response.

Demographics	Univariate Analysis		Multivariate Analysis	
	HR	*p*-Value	HR	*p*-Value
Age (per 1 year increase)	1.03 [0.99; 1.06]	0.09		
Gender Female Male	Reference 1.43 [0.52; 3.96]	0.48		
**Clinical characteristics**				
Performance status 0	Reference			
1	1.45 [0.42; 4.95]	0.55		
2	0.89 [0.16; 4.85]	0.89		
3	4.67 [0.85; 25.69]	0.07		
AJCC 8th stage M1A/IV	Reference			
M1B/IV	2.80 [0.54; 14.47]	0.22		
M1C/IV	0.73 [0.24; 2.22]	0.57		
M1D/IV	0.00 [0.00; Inf]	0.99		
Total RT dose	1.02 [0.98; 1.06]	0.27		
RT dose per fraction	1.10 [0.98; 1.24]	0.11		
RT duration	0.96 [0.90; 1.04]	0.34		
Number of irradiated metastases One metastasis Multiple metastasis (≥2)	* Reference 3.16 [1.12; 8.89]	**0.03**	16.85 [2.16; 131.49]	**<0.01**
Irradiated metastases				
Abdomen (spleen, liver)	0.20 [0.01; 3.27]	0.26		
Lymph node	0.52 [0.06; 4.35]	0.55		
Skin	0.56 [0.06; 4.83]	0.60		
Bone	0.00 [0.00; Inf]	0.99		
Lung	1.56 [0.14; 17.29]	0.72		
Breast	0.00 [0.00; Inf]	0.99		
Muscle	0.00 [0.00; Inf]	0.99		
Immunotherapy None Concomitant with RT	Reference 7.56 [0.99; 57.57]	**0.05**		
Ipilimumab	Reference			
Nivolumab	2.05 [0.26; 16.21]	0.49		
Nivolumab + ipilimumab	3.79 [0.34; 41.86]	0.28		
Pembrolizumab	1.32 [0.12; 14.59]	0.82		
Occurrence of immune-related events *	64.83 [14.48; 290.18]	**<0.01**	403.45 [13.83; 11769.39]	**<0.01**

AJCC 8th stage = American Joint Committee on Cancer 8th edition; RT = radiotherapy; immune-related events * = episodes of infection and symptoms of acute clinical inflammation (redness, heat, swelling, pain, loss of function, and/or biological inflammatory syndrome with the elevation of inflammation parameters, including, at least, C-reactive protein).

**Table 7:** ROC analysis.

	Total Radiation Dose (Gray)	Dose per Session (Gray)	Fractionation of Radiation	Total Treatment Time, Including Breaks (Days)
Optimal value	19	5.25	3.5	4.5
Specificity	0.765	0.679	0.691	0.864
Sensitivity	0.8	1	0.8	0.8

## Data Availability

The data presented in this study are available on request from the corresponding author.

## Abbreviations

AJCC 8th	American Joint Committee on Cancer 8th edition AJCC 8th
AUC	Area Under the curve
AR	Abscopal Response
CR	complete response
CRP	C-Reactive Protein
CTCAE v5	Common Terminology Criteria for Adverse Events version 5
CTLA-4	cytotoxic T lymphocyte antigen
EBRT	external beam radiation therapy
18F-FDG-PET/CT	18F-Fluorodeoxyglucose positron emission tomography with CT
HR	hazard ratio
ICI	immune checkpoint inhibitors
LC	local control of the irradiated lesion
MM	Metastatic melanoma
OS	Overall survival
PD-1	programmed cell death 1
PD-L1	programmed cell death-ligand 1
PFS	progression-free survival
PTV	planning target volume
PR	partial response
RECIST	Response Evaluation Criteria in Solid Tumors
RT	Radiotherapy
SBRT	Stereotactic body radiation therapy
